# Isolation of fungi from dead arthropods and identification of a new mosquito natural pathogen

**DOI:** 10.1186/s13071-016-1763-3

**Published:** 2016-09-05

**Authors:** Sana Jaber, Alex Mercier, Khouzama Knio, Sylvain Brun, Zakaria Kambris

**Affiliations:** 1 Biology Department, American University of Beirut, Beirut, Lebanon; 2 Equipe Genetique et Epigenetique des Champignons, LIED, UMR8236, Universite Paris-Diderot, Paris, France

**Keywords:** Fungi, Entomopathogen, Vector control, *Beauveria bassiana*, *Drosophila*, *Aedes*, Infection

## Abstract

**Background:**

Insects are well known vectors of human and animal pathogens and millions of people are killed by mosquito-borne diseases every year. The use of insecticides to target insect vectors has been hampered by the issues of toxicity to the environment and by the selection of resistant insects. Therefore, biocontrol strategies based on naturally occurring microbial pathogens emerged as a promising control alternative. The entomopathogenic fungus *Beauveria bassiana* is well characterized and have been approved by the United States Environmental Protection Agency as a pest biological control method. However, thousands of other fungi are unexploited and it is important to identify and use different fungi for biocontrol with possibly some vector specific strains. The aim of this study was to identify new fungal entomopathogens that may be used as potential mosquito biocontrol agents.

**Methods:**

Cadavers of arthropods were collected from pesticide free areas and the fungi associated isolated, cultured and identified. Then the ability of each isolate to kill laboratory insects was assayed and compared to that of *B. bassiana*.

**Results:**

In total we have isolated and identified 42 fungal strains from 17 different arthropod cadavers. Twenty four fungal isolates were cultivated in the laboratory and were able to induce sporulation. When fungal spores were microinjected into *Drosophila melanogaster*, eight isolates proved to be highly pathogenic while the remaining strains showed moderate or no pathogenicity. Then a selection of isolates was tested against *Aedes* mosquitoes in a model mimicking natural infections. Only one fungus (*Aspergillus nomius*) was as pathogenic as *B. bassiana* and able to kill 100 % of the mosquitoes.

**Conclusion:**

The obtained results are encouraging and demonstrate the feasibility of this simple approach for the identification of new potential mosquito killers. Indeed, it is essential to anticipate and prepare biocontrol methods to fight the expansion of mosquitoes’ habitat predicted in certain geographical areas in association with the occurring climatic changes.

**Electronic supplementary material:**

The online version of this article (doi:10.1186/s13071-016-1763-3) contains supplementary material, which is available to authorized users.

## Background

Insects are an essential component of all ecosystems. However, they can be detrimental to crop production and more dramatically many insects are disease-vectors for plants (herbivores) and for animals (blood-feeding). Mosquitoes for instance, are vectors of several deadly human diseases like malaria, dengue, chikungunya and more recently emerging Zika [1–4]. Billions of human lives are threatened by mosquito-borne diseases especially in tropical and sub-tropical zones. In Lebanon, several mosquito species are present, some of which are known to be vectors of disease including mosquitoes of the *Culex* group and the Asian tiger mosquito *Aedes albopictus* [5]. Climate warming may lead to the spread of mosquito-borne diseases in the near future. Indeed, *A. albopictus* occurrence was reported for the first time in Lebanon about 10 years ago and its population size and geographical distribution has considerably increased since then. Therefore several factors place Mediterranean countries at an increased risk of epidemics [6]. The recent reports of patients infected with chikungunya virus (spread by *Aedes* mosquitoes) in the south of France are an example [7]. The frequent travel and massive mobility of people associated with modern life and the presence of endogenous mosquito vectors places some areas such as the Mediterranean countries at high risk of epidemics.

The use of insecticides to target mosquitoes has been hampered by the issues of environmental contamination and risks for human health and by the emergence of resistance problems [8]. Therefore, biocontrol strategies are desirable, and the use of the endosymbiotic bacterium *Wolbachia* has been proposed as a possibility [9–12]. Also, control strategies based on naturally occurring microbial pathogens emerged as another promising alternative to control insects. Fungi are the most common and the most studied cause of insect disease in nature and approximately 1000 fungal species are reported to kill insects, aphids, mites etc. [13]. Spores of the fungus *Beauveria bassiana* have been approved by the United States Environmental Protection Agency as a pest biological control method.

Several laboratory studies have shown that insects are sensitive to infections with *B. bassiana* and this fungus is commonly used to study insect immunity particularly in the model organism *Drosophila melanogaster* [14]. The genetic dissection of the regulation of the immune response in *Drosophila* and in mosquitoes has made a breakthrough in the understanding of the innate immune system in Diptera but also in mammals [15]. Insects depend only on innate defences to fight pathogens: the process is initiated when microbial determinant called Pathogen-Associated Molecular Patterns (PAMPs) are recognized by the host Pattern Recognition Receptors (PRRs) leading to the activation of genetic pathways and triggering effector responses. These studies have shown that despite the fact that these immune reactions are innate, they are adapted to the type of invading pathogen [14]. Anti-fungal responses depend on the detection of fungal cell wall components by the PRR GNBP3 and on a parallel pathway involving the serine protease Persephone that needs to be processed by secreted fungal proteases to activate the Toll pathway [16]. Although entomopathogenic fungi are widespread within the Eumycetes, the focus has been put on Ascomycota and in particular the family of the Chordicipitaceae with its two representative genera *Metharyzium* and *Beauveria* [17–19]. Therefore, most of the data collected in *Drosophila* rely on challenge with *B. bassiana* as a fungal model, and infections with other entomopathogenic fungi may be needed to identify additional host response molecules.

Since both *Metharizium* and *Beauveria* are well characterized, the presence of their spores on the cuticle often serves as a visual indicator during the collection of insect cadavers. Hence, many studies report isolates of already known species of both fungi and most of them are stocked in the ARSEF collection of the US Department of Agriculture (USDA) [20]. An example is the isolation of a *Beauveria* species in an attempt aiming to identify a natural killer of the invasive sawfly *Cephalcia tannourinensis* which infests Cedar trees in Lebanon [21]. In the present study, the aim was to identify new fungal entomopathogens that may be used as potential mosquito biocontrol agent.

## Methods

### *Drosophila* and mosquito strains

*Drosophila melanogaster W*^*1118*^ strain was used in infection experiments as wild-type flies*.* Stocks were reared in 50 ml vials containing standard cornmeal agar food prepared according to the Drosophila Bloomington Stock Center recipe. Flies were kept at 24 °C, 45 % relative humidity on a 12 h light/dark photocycle. *Aedes albopictus* (Sarba strain) a local mosquito strain was reared in the insectary at 28 °C, 70 % relative humidity on a 12 h light/dark photocycle. Mosquito cages were supplemented with a cup of tap water and a cotton pad soaked in 10 % sucrose. Eggs were collected 4 days after a blood meal and allowed to air dry for 2 weeks before hatching. Dried eggs were hatched by immersion into deoxygenated water. Larvae were reared in pans containing tap water and fed on beer-brewing yeast for the first day after hatching then on fish pellets till pupation.

### Fungus strain

*Beauveria bassiana* strain 80.2 (a gift from Dominique Ferrandon) was used as a control in all survival experiments.

### Arthropod cadaver collection

Two series of dead animals were analyzed: the first series was collected in July 2014 from the American University of Beirut campus; the temperature range was 27–32 °C and relative humidity of 70–80 %. The second series was obtained in May 2015 from Nabatieh area (south of Lebanon); the temperature range was 20–26 °C and humidity around 70 %. Areas where insecticides may have been used were avoided and cadavers in the vicinity of spider nets or incandescent lights were also disregarded.

### Fungus isolation

Carcasses were suspended in water containing 5 % Tween and shaken vigorously to resuspend spores or mycelium fragments present on the cuticle surface. Ten μl of different dilutions of this suspension was plated on standard PDA/chloramphenicol medium. After one or two days incubation at 27 °C, individual germinations (or mycelium regeneration) were transferred to a new plate. For each insect, only one isolate per group of morphologically identical thalli was selected. These isolates were submitted to several rounds of purification in order to follow morphological stability after the successive transfers. Conidial species such as *Aspergillus* spp. and *Penicillium* spp. were submitted to single spore purification.

### DNA extraction and sequencing

Fungal isolates were grown on cellophane/PDA for two to four days at 27 °C. DNA was extracted as in [22]. ITS sequences were PCR amplified with the following universal primers: ITS1 (5'- TCC GTA GGT GAA CCT GCG G-3') and ITS4 (5'-TCC TCC GCT TAT TGA TAT GC-3'). Amplification and direct sequencing of fungal ribosomal RNA genes was as in [23]. Sequences (Additional file 1: Table S1) were blasted against NCBI GenBank for identification purposes [24].

### Spore purification

Fungal spores were extracted from four week-old PDA plates by adding 25 ml sterile distilled water to each plate and scrapping the surface. A sterile funnel containing autoclaved glass wool was used to separate the spores from other mycelia structures. The collected spore suspension was centrifuged at 4000× *g* and washed three times with distilled water and finally resuspended in 0.5 ml water. Spores were then counted using a hemocytometer and diluted to the desired concentration. Freshly prepared fungal spore solutions were used for all *Drosophila* and mosquito challenges.

### Infection and survival assays

Survival experiments were performed on batches of 15 wild-type flies or 20 mosquitoes. In all experiments, 3 to 7 day-old females were used. Mosquitoes were only sugar-fed (no blood meal). Two types of infection were performed: microinjections and infections by spraying. For microinjections, flies were anesthetized on a CO_2_ flow bed and 32 nl of water containing 100 fungal spores were injected into the thorax using a NanodropII microinjector (Drummond Scientific, California, USA). For infections by spraying, a suspension of 50 × 10^6^ spores/ml was sprayed on anesthetized mosquitoes. Vials (for *Drosophila melanogaster*) or cups (for *Aedes* spp.) containing the challenged animals were then put in an incubator at 29 °C and the surviving flies counted every few hours. Flies that died within the first 2 h after injection were disregarded since their death is considered to be due to the needle injury. Each experiment was repeated at least three independent times, and a representative result is shown.

### Statistical analysis

For statistical analysis of the survival data, Gehan-Breslow-Wilcoxon test was performed. Results with a *P*-value of less than 0.05 were considered as significant. Detailed analysis report is provided in Additional file 2: Table S2.

## Results

### Dead arthropod collection and identification

Dead arthropod identification was based on morphological criteria and determined to lowest taxonomic rank possible. Depending on the preservation of the specimen, the size of its group and the presence of distinctive features, variable rank levels could be determined with confidence for different animals. Dead arthropods 1 to 6 were collected in Beirut, and cadavers 7 to 17 were sampled from a more rural area in the south of Lebanon. Specimens collected were from the orders Coleoptera, Lepidoptera, Hemiptera, Hymenoptera, Thysanura, Isopoda, Aranea, Polydesmida and Diptera (see Table 1 and Additional file 3: Figure S1).

**Table 1:** List of the collected dead arthropods and the corresponding fungi. Fungus # refers to the arthropod it was isolated from and letters correspond to different fungal isolates. Arthropod order is given in parentheses. The last column summarizes the results of spore microinjection: + denotes a pathogenic fungus (killing *Drosophila* with no statistically significant difference than *B. bassiana*, *P* > 0.05); − denotes a mildly pathogenic or non-pathogenic fungus (killing at a statistically significant different rate compared to *B. bassiana*, *P* < 0.05)

Fungus #	Fungus species	Carrier arthropod	Pathogenicity
1a	*Aspergillus ustus*	Buprestidae (Coleoptera)	+
1b	*Aspergillus candidus*	Buprestidae (Coleoptera)	–
1c	*Aspergillus sclerotium*	Buprestidae (Coleoptera)	+
1d	*Aspergillus candidus*	Buprestidae (Coleoptera)	–
1e	*Aspergillus nomius*	Buprestidae (Coleoptera)	+
1f	*Aspergillus sclerotium*	Buprestidae (Coleoptera)	+
2a	*Wallemia* sp.	*Culex* sp. Culicidae (Diptera)	+
3a	*Aspergillus sclerotium*	Curculionidae (Coleoptera)	nt
3b	*Scopulariopsis brevicaulis*	Curculionidae (Coleoptera)	–
3c	*Aspergillus sclerotium*	Curculionidae (Coleoptera)	nt
4a	*Aspergillus fumigatus*	Dermestidae (Coleoptera)	nt
4b	*Aspergillus ruber*	Dermestidae (Coleoptera)	nt
4c	*Aspergillus ruber*	Dermestidae (Coleoptera)	+
4d	*Aspergillus glaucus*	Dermestidae (Coleoptera)	–
5a	*Chaetomium globosum*	Lepismatidae (Thysanura)	–
6a	*Pyrenophora dictyoides*	Miridae (Hemiptera)	nt
6b	*Fusarim tricinctum*	Miridae (Hemiptera)	nt
7a	*Botrytis cinerea*	*Apis mellifera*, Apidae (Hymenoptera)	nt
7b	*Alternaria alternata*	*Apis mellifera*, Apidae (Hymenoptera)	+
7c	*Fomes fomentarius*	*Apis mellifera*, Apidae (Hymenoptera)	–
8a	*Talaromyces amestolkiae*	Pyrrhocoridae (Hemiptera)	nt
8b	*Cladosporium cladosporioides*	Pyrrhocoridae (Hemiptera)	–
8c	*Stachybotrys chartarum*	Pyrrhocoridae (Hemiptera)	nt
8d	*Ascomycota* sp.	Pyrrhocoridae (Hemiptera)	nt
9a	*Alternaria infectoria*	*Armadillidium vulgare,* Armadillidae (Isopoda)	–
9b	*Cladosporium cladosporioides*	*Armadillidium vulgare,* Armadillidae (Isopoda)	nt
9c	*Simplicillium sympodiophorum*	*Armadillidium vulgare,* Armadillidae (Isopoda)	nt
10a	*Penicillium digitatum*	Polydesmidae (Polydesmida)	–
10b	*Periconia* sp.	Polydesmidae (Polydesmida)	–
11a	*Penicillium freii*	Pyralidae (Lepidoptera)	–
11b	*Talaromyces amestolkiae*	Pyralidae (Lepidoptera)	nt
12a	*Chaetomium nigricolor*	*Aphodius* sp. Scarabaeidae (Coleoptera)	nt
12b	*Chaetomium bostrychodes*	*Aphodius* sp. Scarabaeidae (Coleoptera)	nt
12c	*Engyodontium album*	*Aphodius* sp. Scarabaeidae (Coleoptera)	–
13a	*Penicillim commune*	Araneidae (Araneae)	+
13b	*Phoma herbarum*	Araneidae (Araneae)	–
14a	*Alternaria infectoria*	Sarcophagidae (Diptera)	nt
14b	*Botrytis cinerea*	Sarcophagidae (Diptera)	nt
15a	*Embellisia abundans*	Araneidae (Araneae)	–
16a	*Talaromyces amestolkiae*	*Capnodis tenebrionis,* Buprestidae (Coleoptera)	nt
17a	*Penicillium polonicum*	*Culex* sp., Culicidae (Diptera)	–
17b	*Talaromyces amestolkiae*	*Culex* sp., Culicidae (Diptera)	–

*Abbreviation*: *nt* not tested

### Isolation and identification of fungi from cadavers

In total, from 17 different dead animals, 130 fungal germinations were isolated and purified on PDA plates. The precise identity of the fungal species isolated from dead arthropods was determined by sequencing PCR-amplified Internal Transcribed Spacers (ITSs) and comparing the results to the GenBank database. The list of insects collected and the corresponding fungi is reported in Table 1. Obtained ITS sequences have been deposited in the GenBank database (accession numbers KX394525–KX394566). In a first step, fungi were clustered according to the morphology of their mycelium. Two morphologies were overrepresented and present on several cadavers. The decision was made to sequence one isolate per cadaver for the overrepresented fungi. The genus *Cladosporium* represented 46 isolates and was found on 12 cadavers (Additional file 1: Table S1). The two other most represented genera were *Penicillium* and *Talaromyces*, two very close genera belonging to the order Eurotiales. *Talaromyces* was isolated 20 times and from four different arthropods. One isolate per insect was sequenced and only one species, *Talaromyces amestolkiae*, was identified. Four morphological groups of *Penicillium* were identified; sequencing revealed that they belong to four different species. *Penicillium commune* was isolated from seven cadavers, *P. digitatum* and *P. frei* from two dead animals each. Except for the above mentioned genera for which a selection has been made in order to avoid unnecessary multiple sequencing of the same species, ITSs were PCR-amplified and sequenced from all of the other isolated and purified fungi. All the fungi that were isolated belong to the *Dikaria* group. Two isolates were basidiomycetes, *Fomes fomentarius* and *Wallemia* sp. The remaining species were ascomycete fungi belonging to the most prevalent phyla Dothideomycetes, Eurotiomycetes, Leotiomycetes and Sordariomycetes. Many of the isolated fungi were saprophytes, others had a life style depending on plants. Interestingly, two fungi, *Simplicillium sympodiophorum* isolated from dead woodlice *Armadillidium vulgare*, and *Engyodontium album* isolated from *Aphodius* sp. (Coleoptera) belong to the *Cordycipitaceae*, a family comprising the genera of the best studied entomopathogens *Metharizium*, *Chordyceps* and *Beauveria*.

### Spore microinjection into *Drosophila* and survival analysis

**Fig. 1:**
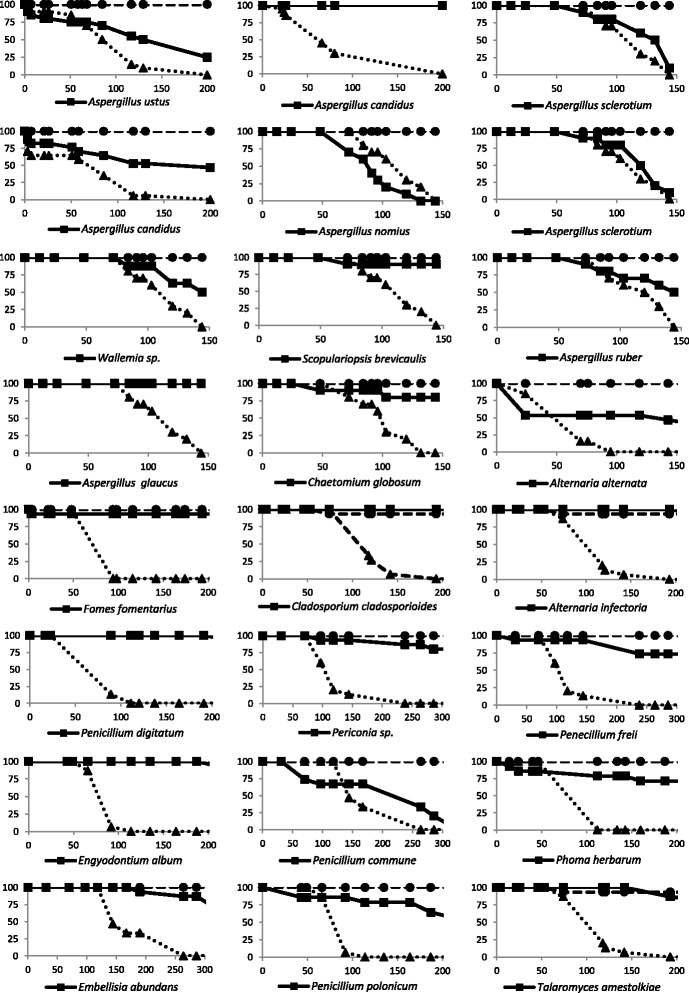
*Drosophila* susceptibility to the microinjection of spores obtained from the different isolated fungi. Survival of *Drosophila* following microinjection of fungal spores (plain line with squares) is shown as percentage of flies alive plotted versus time in hours. In each experiment flies microinjected with the same number of *B. bassiana* spores were used as a reference (dotted line with triangles). In parallel flies microinjected with water are included as control (dashed line with circles). Seven fungi (*A. ustus*, *A. sclerotium*, *A. nomius*, *Wallemia* sp., *A. ruber*, *A. alternata* and *P. commune*) showed pathogenicity levels that were not statistically different compared to those triggered by *B. bassiana* (*P* > 0.05)

After identification, a total of 24 fungal isolates were grown in the laboratory and were able to induce sporulation. Spores were collected, washed, counted and microinjected into wild-type *Drosophila* to determine the pathogenic potential of each isolate. In parallel, for each experiment, the same number of spores obtained from the well characterized entomopathogen *B. bassiana* was microinjected as a reference. Fungi that significantly differed from *B. bassiana* in the rate at which they kill the flies (*P* < 0.05) were considered negatives; these were 16 isolates corresponding to *Aspergillus candidus* (2 isolates), *Scopulariopsis brevicaulis, Aspergillus glaucus, Chaetomium globosum, Fomes fomentarius, Cladosporium cladosporioides, Alternaria infectoria, Penicillium digitatum, Periconia sp., Penicillium freii, Engyodontium album, Phoma herbarum, Embellisia abundans, Penicillium polonicum, Talaromyces amestolkiae*. Among these isolates 13 did not kill more than 25 % of the injected flies while two isolates (*P. herbarum* and *P. polonicum*) killed about 30 % and one isolate (*A. candidus*) killed about 50 %. The isolates that killed with a rate that is not statistically different than that observed with *B. bassiana* (*P* > 0.05) were considered positives. Eight fungi correspond to this category: *Aspergillus ustus, Aspergillus sclerotium* (2 distinct isolates tested)*, Aspergillus nomius, Wallemia sp., Aspergillus ruber, Alternaria alternata and Penicillium commune*. Among these, five isolates (*A. ustus, Wallemia sp. A. ruber*, *A. alternata* and *P. commune*) killed between 50 and 75 % of the injected animals, while only three isolates (*A. nomius* and *A. sclerotium*) were able to kill 100 % of the injected flies. It was noted that *A. nomius* was the only fungus that was able to kill injected *Drosophila* at an even faster rate than *B. bassiana*. These results are shown in Fig. 1 and summarized in Table 1.

### Spore microinjection into *Aedes* spp. and survival analysis

**Fig. 2:**
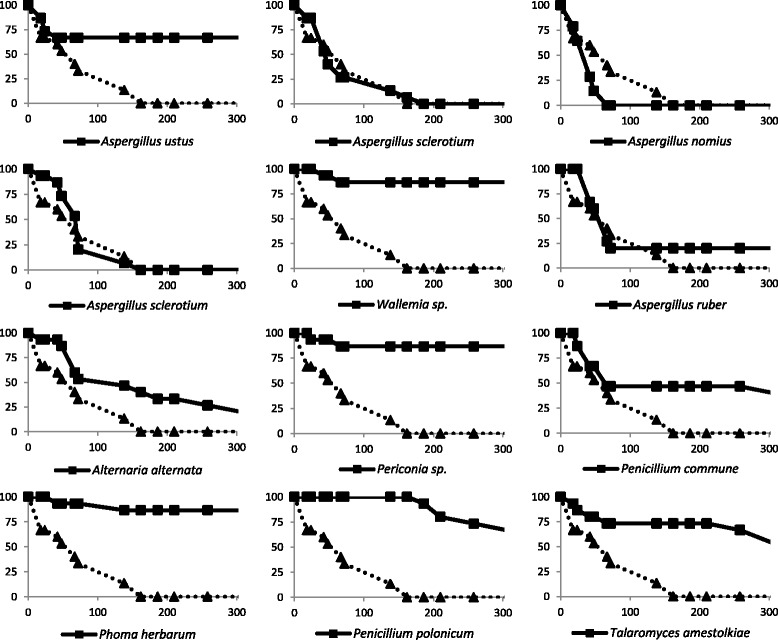
*Aedes* susceptibility to the microinjection of fungal spores**.** Survival of *Aedes* spp. following microinjection of fungal spores is shown. In each experiment flies microinjected with *B. bassiana* spores were used as a reference (dotted line with triangles). *Aedes nomius*, *A. sclerotium* (2 isolates) and *A. ruber* showed death rates that were not statistically different compared to those triggered by *B. bassiana* (*P* > 0.05) indicating that these four isolates are highly pathogenic to *Aedes* spp. Although *A. ustus*, *Wallemia* sp., *A. alternata* and *P. commune* injections led to the death of some injected mosquitoes, the results were statistically different when compared to *B. bassiana* (*P* < 0.05) reflecting low pathogenicity. *Periconia* sp., *P. herbarum, P. polonicum* and *T. amestolkiae* were not highly pathogenic to mosquitoes

Based on these results, a subset of the fungal isolates (including the 8 that were considered positive and four of the isolates that were not highly pathogenic to *Drosophila*) was used to microinject *Aedes* spp. mosquitoes under similar conditions. *Aspergillus nomius, A. sclerotium* (2 isolates) and *A. ruber* showed pathogenicity levels that were not statistically different compared to those triggered by *B. bassiana* (*P* > 0.05) corroborating the results obtained using *Drosophila*. Indeed, *A. ruber* killed about 75 % of injected mosquitoes and *A. nomius, A. sclerotium* led to a 100 % lethality in *Aedes* spp. (Fig. 2). On the other hand, *Periconia* sp., *P. herbarum*, *P. polonicum* and *T. amestolkiae* were not highly pathogenic to mosquitoes in agreement with what has been observed in Fig. 1. However, although *A. ustus, Wallemia* sp., *A. alternata* and *P. commune* injections led to the death of some injected mosquitoes, these isolates were not as pathogenic for *Aedes* spp. as they were for *Drosophila* (Fig. 2).

### Spore spraying onto *Aedes* spp. and survival analysis

**Fig. 3:**
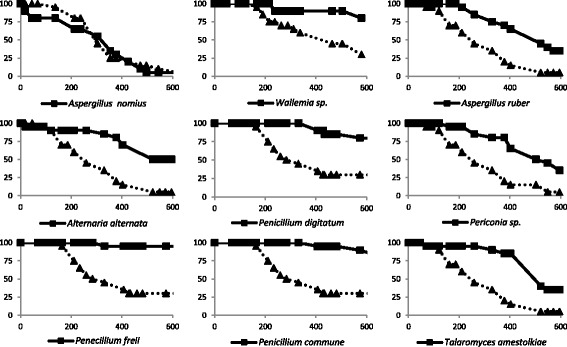
Survival of *Aedes albopictus* mosquitoes after infection by spraying the insects with a suspension of fungal spores (plain line with squares) is shown as the percentage of mosquitoes alive plotted versus time in hours. In each experiment the same number of *B. bassiana* spores was sprayed on control mosquitoes as a reference (dotted line with triangles). Only *A. nomius* was able to kill the mosquitoes at a very similar rate compared to *B. bassiana*. None of the mosquitoes that were mock-sprayed with water under the same conditions succumbed to the treatment (not shown)

**Fig. 4:**
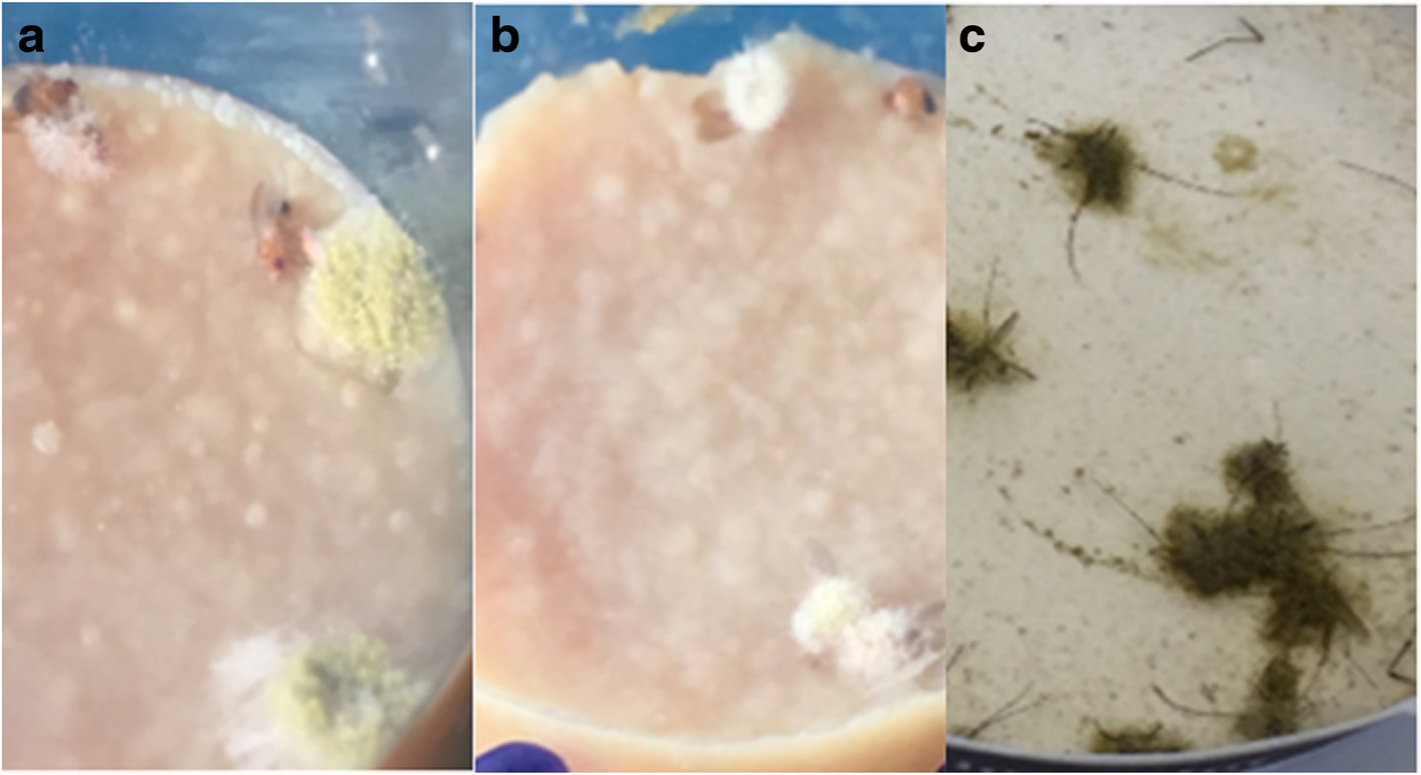
Photos of dead insects after microinjection or spraying with *A. nomius* spores. **a** *Drosophila* cadavers following *A. nomius* spores microinjection. **c** *Aedes* mosquitoes after spraying with the same fungus. The dead insects are completely covered by fungal growth indicating that the cause of death is the development of the spores within the animal. *Drosophila* cadavers after *B. bassiana* spores microinjection are shown for comparison (**b**)

The fact that an isolate showed high virulence in the microinjection experiment does not imply that it is a natural pathogen of insects. Indeed, the insect cuticle is an important barrier that needs to be breached by the germinating fungal spores. Therefore, before concluding that a fungus is a real entomopathogen, it is important to test it in a system that is close to natural infection setting. This can be achieved by spraying spores on the insects without injuring the cuticle. For this reason, we wanted to assay the pathogenicity of *A. nomius* - along with a selection of other isolates - in comparison to *B. bassiana* after spore spraying*.* In this experiment, *A. albopictus* mosquitoes were used as model. Among nine isolates tested with this mode of infection (including *A. nomius*, *Wallemia* sp., *A. ruber*, *A. alternata* and *P. commune* of the fungi that were pathogenic by microinjection and *P. digitatum*, *Periconia* sp., *P. freii* and *T. amestolkiae* of the ones that were not highly pathogenic by microinjection) *A. nomius* was the only fungus that killed at a very similar rate compared to *B. bassiana* (Fig. 3). Interestingly, only in the case of *A. nomius* (in addition to *B. bassiana*), irrespectively of whether the exposure to the spores was by microinjection or via spraying, the dead flies were completely covered by fungal mycelia confirming that the cause of death is due to the development of the spores in the insect and that the spores were able to germinate and probably pierce the mosquito cuticle (Fig. 4). Infection by spraying was also performed with *A. nomius* using another mosquito (*Culex pipiens*) and the results confirmed that this isolate is as pathogenic as *B. bassiana* to mosquitoes (Additional file 4: Figure S2).

## Discussion

In the aim of recovering novel entomopathogens, we isolated fungi from dead arthropods and a subset of isolates per cadaver underwent ITS-sequencing and identification as well as pathogenicity testing. Several isolated fungi are likely to be airborne contaminant and/or saprotrophic fungi that may have developed on the arthropod carcass after the death of the animal has occurred. Examples of such possible contaminants are *Penicillium*, *Talaromyces* and *Cladosporium* isolates that have been oversampled in the course of the survey. *Fomes fomentarius* is known for its role in wood decay and for causing white-rot in plants according to some reports [25]; it has also been used in traditional medicine mostly for its anti-inflammatory and pain-killing properties [26–28]. *Wallemia* sp. has a saprophyte life-style and has been shown in some cases to be involved in food spoilage [29, 30].

Noteworthy, two human-related fungi were isolated in our study: *Aspergillus fumigatus* and *S. brevicaulis. Aspergillus fumigatus* is considered an opportunistic human-pathogen. However, it is primarily a ubiquitous saprophyte fungus present in many natural environments [31]. Although aspergilli are well-known airborne contaminants or soil inhabitants, *A. nomius* proved to be of considerable interest in our survey. Indeed, this fungus was as pathogenic as *B. bassiana* both by microinjection into *Drosophila* and *A. albopictus* or by infection via spore spraying onto *A. albopictus* and *C. pipiens*. Moreover, a study focusing on stonebrood, a fungi-caused disease that affects honey bee larvae, has detected the presence of *A. nomius* in affected hives. Indeed, among the ten *Aspergillus* species identified in honey bee hives, *A. flavus*, *A. phoenicis* and *A. nomius* were shown to be pathogenic to the larvae [32].

In the present study, *A. nomius* was isolated from a dead beetle (Coleoptera: Buprestidae) and was able to develop on and kill both *Drosophila* and mosquitoes (Diptera) indicating that it is a general entomopathogen with a broad host range. Targeting different insects can be considered an advantage, since the same fungus can be used to target several pests. However, fungi with a broad range of target insects can lead to the undesirable killing of non-target species and they should be used with caution [33, 34]. In contrast, bacteria can be used to kill insects in a very specific manner, due to the presence of toxin receptors on their epithelial cells in the target species [35].

Host-range specificity could also be correlated to differences in the immune systems of the target insects. Therefore, in addition to a potential use as biocontrol agent, *A. nomius* could be used as elicitor of insect immune responses in model organisms to decipher the pathways involved in the recognition of fungal infections. Indeed, although the major antifungal players have been characterized such as GNBP3 which plays different roles, both activating Toll pathway and assembling effector complexes that directly attack fungi, some aspects of insect antifungal responses remain unknown [36, 37].

Differences in the immune system between *Aedes* and *Drosopila* could explain the fact that from the eight fungal isolates that were pathogenic to *Drosophila*, only four (including *A. nomius*) were pathogenic to the mosquito*,* while *A. ustus*, *Wallemia* sp., *A. alternata* and *P. commune* were not as pathogenic to the mosquitoes as *B. bassiana*. It is worth mentioning that these four isolates were relatively “mild” in *Drosophila* (killing between 50 and 75 % of injected flies) as compared to the four that killed both *Drosophila* and *Aedes* (killing 75–100 % of injected flies). However, these differences are not surprising if we take into consideration that even between *Drosphila* species there are differences in antifungal defenses [38].

The mildly pathogenic fungi too can be interesting as biocontrol agent if they show more restricted host range as compared to the virulent ones. Also, the slow killing rate can allow more time for the infected animals to spread the spores within a population, especially because it has been reported that *Anopheles* female mosquito are attracted to dead insect carrying *B. bassiana* spores [39] and because transmission of *B. bassiana* from male to female *Aedes* mosquitoes has been observed [40].

## Conclusions

The identification of *A. nomius* as a new natural insect pathogen and a potential disease-vector control agent is encouraging. More importantly, this pilot study demonstrates the feasibility of a simple approach for the identification of potential mosquito killers, especially that this may provide a solution to pest control from within the ecosystem rather than utilizing toxic substances. Indeed, it is essential to anticipate and prepare biocontrol methods to fight the expansion of mosquitoes’ habitat predicted in certain geographical areas in association with the occurring climatic changes. A larger scale screen could be conducted in the aim of identifying more entomopathogens with perhaps some fungi that are specific to certain host families and to give a more precise idea about saprophyte fungi that decompose arthropod cadavers in nature.

## Additional files

Additional file 1: Table S1. Number of occurrences of each isolated fungus in the collected cadavers. (DOCX 22 kb) Additional file 2: Table S2. Statistical analysis for survival experiments. (DOCX 23 kb) Additional file 3: Figure S1. Photos of the dead arthropods from which fungi were isolated. (DOCX 505 kb) Additional file 4: Figure S2. Effects of *A. nomius* on another species of mosquitoes: *Culex pipiens.* (PPTX 90 kb)

## Abbreviations

PCR: Polymerase chain reaction
PDA: Potato dextrose agar
