# Supplementary material for: Isolation of fungi from dead arthropods and identification of a new mosquito natural pathogen
Source: Parasit Vectors. 2016 Sep 5;9(1):491. doi: 10.1186/s13071-016-1763-3 (PMC5012000; doi:10.1186/s13071-016-1763-3)
Supplement: Additional file 1: Table S1. — Number of occurrences of each isolated fungus in the collected cadavers. (DOCX 22 kb) [file 13071_2016_1763_MOESM1_ESM.docx]

**Additional file 1: Table S1: Number of occurrences of each isolated fungus among the collected cadavers.**

In this table each isolated fungus is listed, its class and known mode of life are given in columns 2 and 3 respectively. A + sign indicates that the fungus was present on the cadaver of a given arthropod (arthropods ID s are given as in table 1).

| **Species** | **Class** | **Mode of life** | **1** | **2** | **3** | **4** | **5** | **6** | **7** | **8** | **9** | **10** | **11** | **12** | **13** | **14** | **15** | **16** | **17** |
| --- | --- | --- | --- | --- | --- | --- | --- | --- | --- | --- | --- | --- | --- | --- | --- | --- | --- | --- | --- |
| *Aspergillus ustus* | Eurotiomycete | Saprophyte | + |  |  |  |  |  |  |  |  |  |  |  |  |  |  |  |  |
| *Aspergillus candidus* | Eurotiomycete | Saprophyte | + |  |  |  |  |  |  |  |  |  |  |  |  |  |  |  |  |
| *Aspergillus sclerotium* | Eurotiomycete | Saprophyte | + |  | + |  |  |  |  |  |  |  |  |  |  |  |  |  |  |
| *Aspergillus nomius* | Eurotiomycete | Saprophyte | + |  |  |  |  |  |  |  |  |  |  |  |  |  |  |  |  |
| *Wallemia* sp*.* | Wallemiomycete | Saprophyte |  | + |  |  |  |  |  |  |  |  |  |  |  |  |  |  |  |
| *Scopulariopsis brevicaulis* | Sordariomycete | Human pathogen |  |  | + |  |  |  |  |  |  |  |  |  |  |  |  |  |  |
| *Aspergillus fumigatus* | Eurotiomycete | Opportunistic pathogen |  |  |  | + |  |  |  |  |  |  |  |  | + |  |  | + |  |
| *Aspergillus ruber* | Eurotiomycete | Saprophyte |  |  |  | + |  |  |  |  |  |  |  |  |  |  |  |  |  |
| *Aspergillus Glaucus* | Eurotiomycete | Saprophyte |  |  |  | + |  |  |  |  |  |  |  |  |  |  |  |  |  |
| *Chaetomium globosum* | Sordariomycete | Saprophyte |  |  |  |  | + |  |  |  |  |  |  |  |  |  |  |  |  |
| *Pyrenophora dictyoides* | Dothideomycete | Plant pathogen |  |  |  |  |  | + |  |  |  |  |  |  |  |  |  |  |  |
| *Fusarium tricinctum* | Eurotiomycete | Saprophyte |  |  |  |  |  | + |  |  |  |  |  |  |  |  |  |  |  |
| *Botrytis cinerea* | Leotiomycete | Plant pathogen |  |  |  |  |  |  | + |  |  |  |  |  |  |  |  |  |  |
| *Alternaria alternata* | Dothideomycete | Plant pathogen |  |  |  |  |  |  | + |  |  |  |  |  |  |  |  |  |  |
| *Fomes fomentarius* | Agaricomycete | Saprophyte |  |  |  |  |  |  | + | + | + |  |  |  |  |  |  |  |  |
| *Talaromyces amestolkiae* | Eurotiomycete | Saprophyte |  |  |  |  |  |  |  | + |  |  | + |  |  |  |  | + | + |
| *Cladosporium cladosporioides* | Dothideomycete | Saprophyte |  |  |  |  |  | + | + | + | + | + | + | + | + | + | + | + | + |
| *Stachybotrys chartarum* | Sordariomycete | Saprophyte |  |  |  |  |  |  |  | + |  |  |  |  |  |  |  |  |  |
| *Ascomycota* sp. | Leotiomycete | Undefined |  |  |  |  |  |  |  | + |  |  |  |  |  |  |  |  |  |
| *Alternaria infectoria* | Dothideomycete | Plant pathogen |  |  |  |  |  |  |  |  | + |  |  |  |  | + |  |  |  |
| *Simplicillium sympodiophorum* | Sordariomycete | Saprophyte |  |  |  |  |  |  |  |  | + |  |  |  |  |  |  |  |  |
| *Penicillium digitatum* | Eurotiomycete | Saprophyte |  |  |  |  |  |  |  |  |  | + |  |  | + |  |  |  |  |
| *Periconia* sp. | Sordariomycete | Saprophyte |  |  |  |  |  |  |  |  |  | + |  |  |  |  |  |  |  |
| *Penicillium freii* | Eurotiomycete | Saprophyte |  |  |  |  |  |  |  |  |  |  | + |  |  |  | + |  |  |
| *Chaetomium nigricolor* | Sordariomycete | Saprophyte |  |  |  |  |  |  |  |  |  |  |  | + |  |  |  |  |  |
| *Chaetomium bostrychodes* | Sordariomycete | Saprophyte |  |  |  |  |  |  |  |  |  |  |  | + |  |  |  |  |  |
| *Engyodontium album* | Sordariomycete | Plant endophyte |  |  |  |  |  |  |  |  |  |  |  | + |  |  |  |  |  |
| *Penicillium commune* | Eurotiomycete | Saprophyte |  |  |  |  |  | + | + |  | + | + |  |  | + | + | + |  |  |
| *Phoma herbarum* | Dothideomycete | Plant pathogen |  |  |  |  |  |  |  |  |  |  |  |  | + |  |  |  |  |
| *Embellisia abundans* | Dothideomycete | Plant pathogen |  |  |  |  |  |  |  |  |  |  |  |  |  |  | + |  |  |
| *Penicillium polonicum* | Eurotiomycete | Saprophyte |  |  |  |  |  |  |  |  |  |  |  |  |  |  |  |  | + |
