# Supplementary material for: Isolation of fungi from dead arthropods and identification of a new mosquito natural pathogen
Source: Parasit Vectors. 2016 Sep 5;9(1):491. doi: 10.1186/s13071-016-1763-3 (PMC5012000; doi:10.1186/s13071-016-1763-3)
Supplement: Additional file 2: Table S2. — Statistical analysis for survival experiments. (DOCX 23 kb) [file 13071_2016_1763_MOESM2_ESM.docx]

**Additional file 2: Table S2: Statistical analysis for survival experiments**

For statistical analysis of all survival data (Figures 1, 2, 3 and S2), F-Test for Variance were calculated and Gehan-Breslow-Wilcoxon test was applied using GraphPad Prism 5 software. Results with a p-value of less than 0.05 were considered as significant.

| **Species** | **Mean** | **Variance** | **F-value** | **p-value** | **df** | **Chi square** |
| --- | --- | --- | --- | --- | --- | --- |
| *Aspergillus ustus* | 85 | 518.2 | 0.4 | 0.0553 | 1 | 3.673 |
| *Aspergillus candidus* | 100 | 0 | 0 | 0.0001 | 1 | 36.39 |
| *Aspergillus sclerotium* | 77.3 | 781.8 | 0.57 | 0.2595 | 1 | 1.271 |
| *Aspergillus candidus* | 72.7 | 278.7 | 0.27 | 0.006 | 1 | 7.543 |
| *Aspergillus nomius* | 52.5 | 1675 | 1.35 | 0.1073 | 1 | 2.594 |
| *Aspergillus sclerotium* | 75 | 990.9 | 0.8 | 0.4194 | 1 | 0.6519 |
| *Wallemia* sp*.* | 85.4 | 307.8 | 0.25 | 0.5679 | 1 | 0.3262 |
| *Scopulariopsis brevicaulis* | 93.3 | 24.2 | 0.02 | 0.0025 | 1 | 9.161 |
| *Aspergillus ruber* | 89.1 | 69.09 | 0.05 | 0.202 | 1 | 1.628 |
| *Aspergillus glaucus* | 100 | 0 | 0 | 0.0001 | 1 | 17.8 |
| *Chaetomium globosum* | 89.2 | 62.9 | 0.04 | 0.0046 | 1 | 8.038 |
| *Alternaria alternata* | 56.7 | 330.2 | 0.19 | 0.5283 | 1 | 0.3977 |
| *Fomes fomentarius* | 93.8 | 3.2 | 0.001 | 0.0001 | 1 | 20.02 |
| *Cladosporium cladosporioides* | 100 | 0 | 0 | 0.0001 | 1 | 28.1 |
| *Alternaria infectoria* | 97.6 | 68 | 0.03 | 0.0001 | 1 | 26.27 |
| *Penicillium digitatum* | 93.3 | 170.9 | 0.1 | 0.0001 | 1 | 28.59 |
| *Periconia* sp. | 91.3 | 59.7 | 0.03 | 0.0001 | 1 | 20.33 |
| *Penicillium freii* | 86 | 123 | 0.06 | 0.0002 | 1 | 13.45 |
| *Engyodontium album* | 99.3 | 4.4 | 0.002 | 0.0001 | 1 | 27.57 |
| *Penicillium commune* | 60 | 967.9 | 0.44 | 0.2788 | 1 | 1.173 |
| *Phoma herbarum* | 81.8 | 85.3 | 0.03 | 0.0008 | 1 | 11.23 |
| *Embellisia abundans* | 94.5 | 78.3 | 0.04 | 0.0001 | 1 | 22.69 |
| *Penecillium polonicum* | 80 | 145.1 | 0.06 | 0.0004 | 1 | 12.66 |
| *Talaromyces amestolkiae* | 96.1 | 100.7 | 0.04 | 0.0001 | 1 | 28.84 |

**Statistical analysis for Figure 1**

| **Species** | **Mean** | **Variance** | **F-value** | **p-value** | **df** | **Chi square** |
| --- | --- | --- | --- | --- | --- | --- |
| *Aspergillus ustus* | 71.3 | 106.6 | 0.09 | 0.0128 | 1 | 6.196 |
| *Aspergillus sclerotium* | 33.8 | 1351.6 | 1.18 | 0.7565 | 1 | 0.0962 |
| *Aspergillus nomius* | 22 | 1253.9 | 1.09 | 0.1648 | 1 | 1.93 |
| *Aspergillus sclerotium* | 40.5 | 1855.3 | 1.62 | 0.4739 | 1 | 0.5128 |
| *Wallemia* sp. | 90.8 | 33.6 | 0.03 | 0.0001 | 1 | 18.57 |
| *Aspergillus ruber* | 45.6 | 1206.3 | 1.09 | 0.5 | 1 | 0.4549 |
| *Alternaria alternata* | 60 | 866.7 | 0.75 | 0.0231 | 1 | 5.163 |
| *Periconia* sp. | 90.3 | 26.8 | 0.02 | 0.0001 | 1 | 18.35 |
| *Penicillium commune* | 60.5 | 470.1 | 0.41 | 0.0414 | 1 | 4.161 |
| *Phoma herbarum* | 91.8 | 30.8 | 0.03 | 0.0001 | 1 | 20.49 |
| *Penecillium polonicum* | 93.3 | 140.7 | 0.11 | 0.0001 | 1 | 27.64 |
| *Talaromyces amestolkiae* | 76.9 | 137.9 | 0.12 | 0.0056 | 1 | 7.67 |

**Statistical analysis for Figure 2**

| **Species** | **Mean** | **Variance** | **F-value** | **p-value** | **df** | **Chi square** |
| --- | --- | --- | --- | --- | --- | --- |
| *Aspergillus nomius* | 48.3 | 1173.8 | 0.75 | 0.4462 | 1 | 0.5803 |
| *Wallemia* sp. | 94.4 | 39.6 | 0.07 | 0.0013 | 1 | 10.34 |
| *Aspergillus ruber* | 79.1 | 666.4 | 0.44 | 0.0002 | 1 | 13.81 |
| *Alternaria alternata* | 80.3 | 345.2 | 0.23 | 0.0013 | 1 | 10.34 |
| *Penicillium digitatum* | 95 | 53.1 | 0.06 | 0.0002 | 1 | 14.35 |
| *Periconia* sp. | 82.8 | 489.9 | 0.35 | 0.0003 | 1 | 13.04 |
| *Penicillium freii* | 97.9 | 6.4 | 0.01 | 0.0001 | 1 | 18.03 |
| *Penicillium commune* | 97.8 | 10.6 | 0.01 | 0.0001 | 1 | 15.99 |
| *Talaromyces amestolkiae* | 80.6 | 662.1 | 0.44 | 0.0001 | 1 | 14.71 |

**Statistical analysis for Figure 3**

| **Species** | **Mean** | **Variance** | **F-value** | **p-value** | **df** | **Chi square** |
| --- | --- | --- | --- | --- | --- | --- |
| *Aspergillus nomius* | 57.6 | 767.7 | 1.54 | 0.0507 | 1 | 3.818 |

**Statistical analysis for Figure S2**
