# Supplementary material for: Isolation of fungi from dead arthropods and identification of a new mosquito natural pathogen
Source: Parasit Vectors. 2016 Sep 5;9(1):491. doi: 10.1186/s13071-016-1763-3 (PMC5012000; doi:10.1186/s13071-016-1763-3)

**Additional file 3: Figure S1: Photos of the dead arthropods from which fungi were isolated**

a Buprestidae (Coleoptera); b *Culex sp*. Culicidae (Diptera); c Curculionidae (Coleoptera); d Dermestidae (Coleoptera); e Lepismatidae (Thysanura); f Miridae (Hemiptera); g *Apis mellifera*, Apidae (Hymenoptera); h Pyrrhocoridae (Hemiptera); i *Armadillidium vulgare*, Armadillidae (Isopoda); j Polydesmidae (Polydesmida); k Pyralidae (Lepidoptera); l *Aphodius Sp*. Scarabaeida (Coleoptera); m Araneidae (Araneae); n Sarcophagidae (Diptera); o Araneidae (Araneae); p *Capnodis tenebrionis*, Buprestidae (Coleoptera).


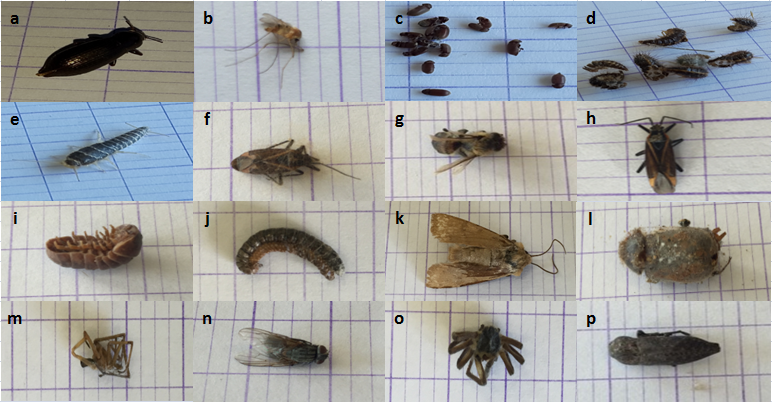

Supplement: Additional file 3: Figure S1. — Photos of the dead arthropods from which fungi were isolated. (DOCX 505 kb) [file 13071_2016_1763_MOESM3_ESM.docx]
