# Supplementary material for: Isolation of fungi from dead arthropods and identification of a new mosquito natural pathogen
Source: Parasit Vectors. 2016 Sep 5;9(1):491. doi: 10.1186/s13071-016-1763-3 (PMC5012000; doi:10.1186/s13071-016-1763-3)
Supplement: Additional file 4: Figure S2. — Effects of A. nomius on another species of mosquitoes: Culex pipiens. (PPTX 90 kb) [file 13071_2016_1763_MOESM4_ESM.pptx]

## Slide 1
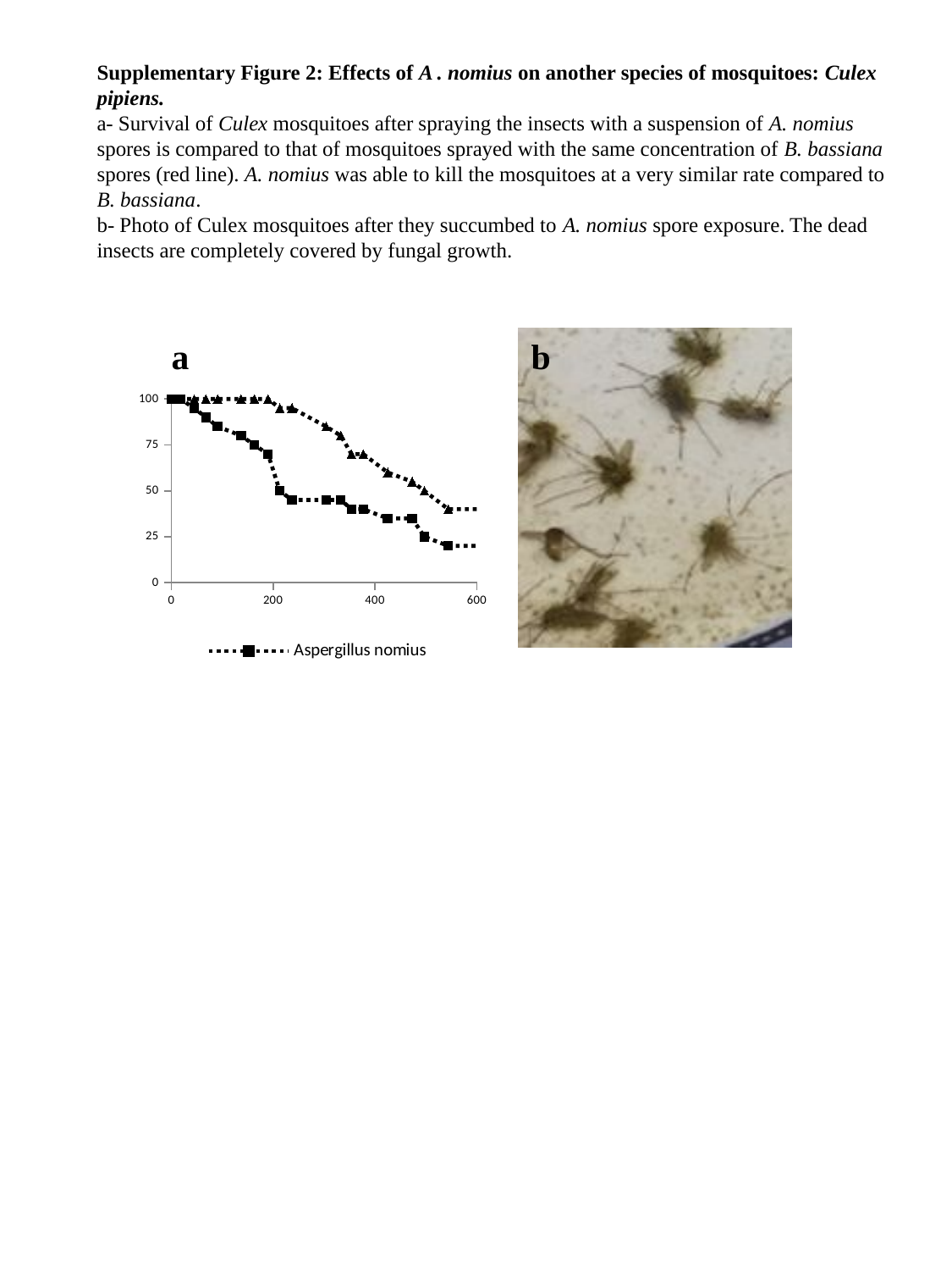

Supplementary Figure 2: Effects of A . nomius on another species of mosquitoes: Culex pipiens.
a- Survival of Culex mosquitoes after spraying the insects with a suspension of A. nomius spores is compared to that of mosquitoes sprayed with the same concentration of B. bassiana spores (red line). A. nomius was able to kill the mosquitoes at a very similar rate compared to B. bassiana.
b- Photo of Culex mosquitoes after they succumbed to A. nomius spore exposure. The dead insects are completely covered by fungal growth.
a
b
### Chart
| Category | Aspergillus nomius | Beauveria bassiana |
|---|---|---|
